# Christmas at the National Sanatorium

**Published:** 1913-01-04

**Authors:** 


### CHRISTMAS AT THE NATIONAL SANATORIUM.

The Christmas festivities at the National Sanatorium, Benenden, were carried out enthusiastically, and the Secretary, Mr. H. Seagrave, assisted by Mr. H. F. Pitt and backed up by the staff, worked very hard. Some 104 of the workers of the country trooped into the large dining hall to a march played on the piano by Mr. C. J. V. Bate, and sat down to tables laden with all the features of an old English Yule feast. The decorations were most effective, both in the recreation hall and the dining hall, whilst various designs and mottoes were hung on the wall, relieved by strings of pennants and flags, many of which were of an ingenious and humorous nature, including the following: " T. B. or no T. B." (alluding, of course, to the tuberculosis bacilli), " Whisper 99," and " Long Life and Good Luck to All." The medical superintendent, Dr. J. C. Smyth, assisted by Dr. G. M. Mayberry, matron, and nurses, waited upon the patients at dinner, tea, and supper, and the patients raised three lusty cheers and finished up by singing " He's a jolly good fellow." Mr. M. J. Mackay performed his famous vanishing trick, and the festivities were continued in the evening of Boxing Day, when the patients held a concert.

